# Pathological Effects of SARS-CoV-2 Associated with Hematological Abnormalities

**DOI:** 10.3390/cimb45090453

**Published:** 2023-08-28

**Authors:** Shama, Asif Mahmood, Shahid Mehmood, Wen Zhang

**Affiliations:** 1 Department of Microbiology, School of Medicine, Jiangsu University, Zhenjiang 212013, Chinaasifuos1@yahoo.com (A.M.); 2 School of Material Science and Engineering, Jiangsu University, Zhenjiang 212013, China; 3 Institute of Life Sciences, Jiangsu University, Zhenjiang 212013, China; shahid.uos45@gmail.com

**Keywords:** SARS-CoV-2, hematology, COVID-19, biomarkers

## Abstract

The SARS coronavirus 2 (SARS-CoV-2) is the causative agent of the 2019 coronavirus disease (COVID-19) pandemic that has claimed the lives of 6.9 million people and infected over 765 million. It has become a major worldwide health problem and is also known to cause abnormalities in various systems, including the hematologic system. COVID-19 infection primarily affects the lower respiratory tract and can lead to a cascade of events, including a cytokine storm, intravascular thrombosis, and subsequent complications such as arterial and venous thromboses. COVID-19 can cause thrombocytopenia, lymphopenia, and neutrophilia, which are associated with worse outcomes. Prophylactic anticoagulation is essential to prevent complications and death rates associated with the virus’s effect on the coagulation system. It is crucial to recognize these complications early and promptly start therapeutic anticoagulation to improve patient outcomes. While rare, COVID-19-induced disseminated intravascular coagulation (DIC) exhibits some similarities to DIC induced by sepsis. Lactate dehydrogenase (LDH), D-dimer, ferritin, and C-reactive protein (CRP) biomarkers often increase in serious COVID-19 cases and poor prognosis. Understanding the pathophysiology of the disease and identifying risk factors for adverse outcomes is critical for effective management of COVID-19.

## 1. Introduction

Severe acute respiratory syndrome coronavirus 2 (SARS-CoV-2) is the causative agent of the COVID-19 (Coronavirus Disease 2019) pandemic, responsible for the incidence of COVID-19 infections globally, which has affected over 765 million people, led to 6.9 million deaths worldwide, and has become a major global health concern [1]. SARS-CoV-2 is related to the Coronaviridae family, which consists of a variety of enveloped RNA viruses of positive sense [2]. SARS-CoV-2 has become the seventh coronavirus that has affected the global population by causing human infection and triggering the worldwide coronavirus 2019 (COVID-19). SARS-CoV-2’s genome sequencing indicated a close relationship to bat coronaviruses, with 96.2% homology. It has a stronger attachment ability for the angiotensin-converting enzyme 2 receptor (ACE-2) to enter host cells and can spread more rapidly than SARS-CoV-2 [3,4]. It is mainly transmitted through close contact or droplets and causes life-threatening disease in 5% of cases, especially in the elderly and in patients with multiple co-existing medical conditions, with a mortality rate of 2.3% worldwide. COVID-19 can cause a wide range of clinical symptoms that can manifest from minor respiratory illness to life-threatening pneumonia, even acute respiratory distress syndrome (ARDS) or organ dysfunction in critical cases, hence adding to the high fatality rate among the SARS-CoV-2-infected population [4,5,6]. Co-factors include the built environment (such as smoking or drug adductors), age, and co-existing diseases (such as arterial hypertension; diabetes mellitus; or liver, lung, and heart disorders). All these factors have a significant role in the mortality rate [7]. Similar to the other two highly virulent coronaviruses that emerged in 2003 with severe acute respiratory syndrome (SARS) and 2012 with Middle East respiratory syndrome (MERS), the most frequent symptoms of COVID-19 observed are fever and respiratory symptoms like cough with sputum and dyspnea [8]. COVID-19 primarily impacts the respiratory tract affected by the SARS-CoV-2 virus, but increasing evidence suggests that it can also affect multiple organs, including the gastrointestinal, neurological, renal, immune, hepatic, and hematological systems. Hematologic manifestations in COVID-19 patients, which have since been widely recognized as having significant prognostic implications, commonly result in venous thromboembolism and related complications, which contribute to increased mortality rates [9]. Autopsy studies have revealed microthrombi in multiple organs, emphasizing the need for effective thromboprophylaxis and treatment. Anomalies in thrombocytopenia and lymphopenia, as well as coagulation panel dysfunctions, are more pronounced among severely affected COVID-19 patients who do not survive. Therefore, early monitoring of hematologic abnormalities is crucial in the diagnosis, prognosis, and management of COVID-19 patients. The aim of this review article is to present a comprehensive overview of the hematological complications associated with COVID-19 with a focus on pathogenesis, biomarkers, and management options.

## 2. Results

**Figure 1:**
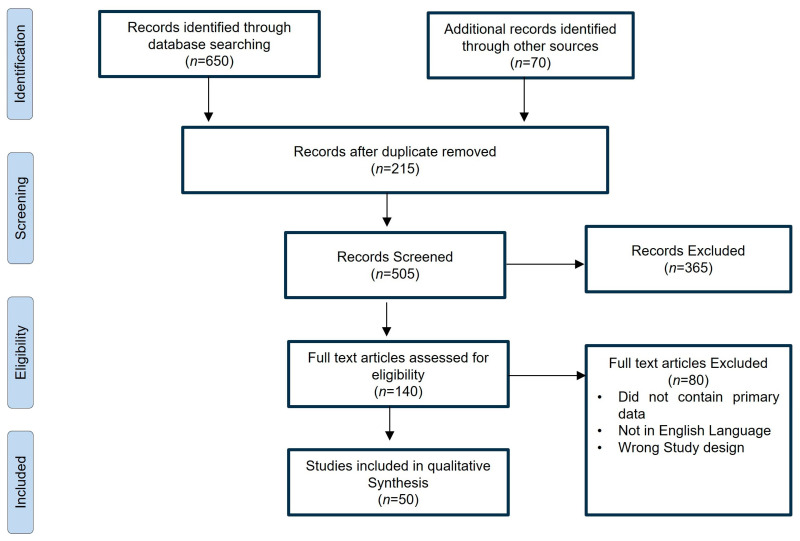
The PRISMA flowchart outlines the literature search process. Initially, 650 articles were identified. After removing duplicates, 505 articles underwent full-text assessment. Ultimately, 50 studies were selected for analysis.

A PRISMA flow chart for the literature search is shown in Figure 1. A total of 650 articles were found. After the removal of duplicates, a total of 505 articles were used for full-text screening, and finally, only fifty studies were included in our analysis.

## 3. COVID-19 and Hematological Abnormalities

The presence of the SARS-CoV-2 virus has been identified as causing COVID-19 with pneumonia-like etiology, and certain studies give much attention to what has been observed, like hematological abnormalities from the SARS-CoV-2 virus [10]. Hematological involvement is widely recognized in coronavirus infections of humans and animals that may vary depending on the strain, species, and intensity of the infection’s virulence [9,11]. Previous studies have discovered that patients with SARS and MERS had differing hematological complications in various studies and populations. However, certain studies have found that thrombocytopenia and lymphopenia are prevalent in a significant number of MERS and SARS patients, with reported rates ranging from 25–90% [12,13]. Moreover, a smaller amount of coagulation abnormalities has also been observed in SARS patients [12].

Although early studies suggested that COVID-19 blood disorder symptoms were uncommon, more recent studies have shown the fact that blood disorder symptoms, including breathlessness, discomfort or chest pain, weakness and fatigue, headache, easy bruising or bleeding, lightheadedness, fast heart beating or heart palpitation, shortness of breath, and swelling of the ankles or legs are present in 30–70% of patients [14,15,16]. In one study in the initial two months of the pandemic outbreak in China, 7736 patients were observed retrospectively to compare the clinical features of severe and non-severe cases. The researcher observed infected individuals with a severe version of the illness were more likely to have low blood counts across all lineages. Among the patients observed, 83.2% had lymphopenia, 33.7% had lymphopenia, and 36.2% had leukopenia. Blood counts showed thrombocytopenia to be the most common abnormality, occurring in 96% of critical cases and 80% of non-critical cases, respectively. In critical cases, the average hemoglobin level has been observed to be lower than in non-critical cases (12.8 g/dL against 13.5 g/dL). Moreover, thrombocytopenia was present in 57.7% of the extremely critical cases and in 31.6% of the less serious cases [5]. Similar findings were found in other observational studies from Chinese patients with COVID-19, although with fewer participants (41, 99, 138, and 201). Lymphopenia was present throughout all categories and was more prevalent in more severe cases [17,18].

In an additional prevalence study, a comparison was made between patients with acute respiratory distress syndrome (ARDS) and those without ARDS. The study included a cohort of 201 individuals with confirmed COVID-19 cases from Hubei province. The findings revealed a significant decrease in lymphocyte and CD-8 T-cell counts among patients with ARDS. Moreover, the study indicated that individuals who exhibited neutrophilia upon infection were at a heightened risk of mortality [19]. Hematological parameters of COVID-19 patients were the focus of descriptive research conducted on 67 individuals at Singapore’s National Centre for Infectious Diseases (NCID). It was found that 29 percent of patients had severe leukopenia, 36.9 percent of patients had lymphopenia, and 5 out of 25 had severe lymphopenia (absolute lymphocyte count [ALC] 0.5 109/L). Seven out of nine patients in the ICU had lymphopenia, and four of them had severe lymphopenia. Twenty percent of their patients also experienced moderate thrombocytopenia (platelet count 100–150 109/L) [20]. This should alert hematologists to be more suspicious of at-risk patients who initially present with signs of a blood problem rather than anticipating respiratory symptoms to develop. It can help in early isolation, detection, and treatment of the COVID-19 virus [21].

Recently, a study by Zhang et al. attempted to describe modifications between COVID-19 patients with hematologic disorders (26/84) and those without hematologic disorders. Despite finding minor differences, in most lab results, higher fibrinogen and D-dimer levels, as well as lower levels of lymphocytes and platelets, were more frequently observed in COVID-19 patients with hematologic disorders than in patients without hematological disorders. Additionally, the study also found that patients with hematological disorders have a high mortality rate and a longer stay in hospitals as compared to those without disorders [22]. Another study also found that COVID-19 patients with hematological disorders had a lower level of lymphocytes and platelets as well as higher levels of ferritin, fibrinogen, and D-dimer and had a higher incidence of ARDS with higher mortality as compared to patients without the disorders [23]. Patients without hematological disorder (malignancies) symptoms are more likely to be discharged home and cured than those with hematological disorder symptoms. This could be due to the outcome and severity of the SARS-CoV-2 virus, which can vary depending on various factors, including immune response, underlying health conditions, patients’ age, and other severe complications, indicating that COVID-19 infected individuals who initially do not present with typical symptoms of the respiratory tract have entered advanced stages of the illness [24] as shown in Table 1. 

## 4. The Hematological System and Its Complications

The pathophysiology of illness caused by COVID-19 contains several organ systems essential for homeostasis [31]. An excessive inflammatory reaction to SARS-CoV-2 directly leads to the release of endogenous compounds favoring altered vascular hemostasis [32]. The liberation of procoagulant and proinflammatory cytokines has a direct effect on blood coagulation [33], thereby triggering the development of systemic coagulation and thromboembolic states, causing widespread damage to the body tissues and organs, especially those most sensitive to ischemic processes tissues and organs such as the cardiovascular system, brain vessels, and lung tissues [34].

### Complications in the Hematological System and Their Mechanism

**Figure 2:**
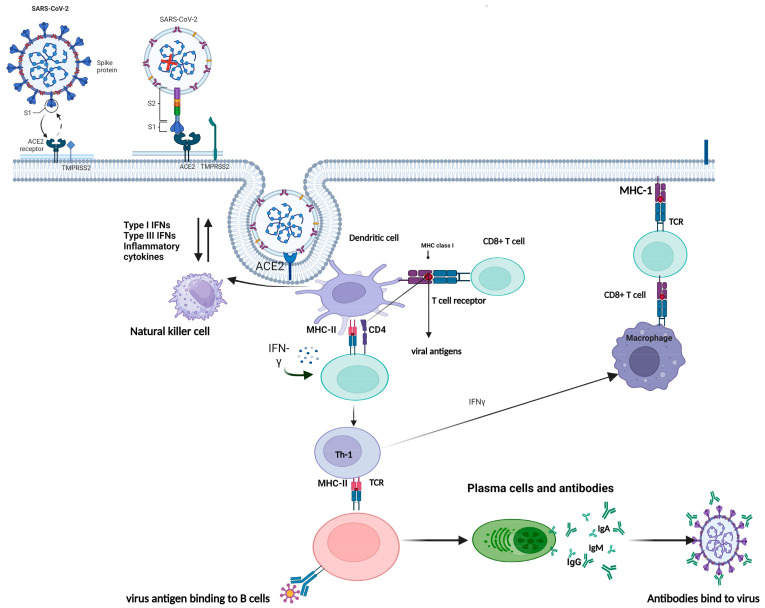
Mechanism of SARS-CoV-2. The mechanism of infection of SARS-CoV-2 is comparable to other coronaviruses, involving the S protein’s interaction with ACE2 (step 1). The S protein comprises the S1 subunit and S2 subunits, with the former containing an RBD (receptor binding domain) that binds to ACE2 receptor and the latter possessing the membrane fusion machinery facilitating cellular entry. Binding of ACE2 to S1 results in conformational changes in the protein, which exposes cleavage sites for proteases like TMPRSS2 or furin located in the cell membrane. This protease activity leads to the separation of S1 and S2 (step 2), activating the membrane fusion machinery of S1 (step 3) and enabling virus entry via endocytosis. Following cellular entry, the virus follows a similar RNA virus replication cycle.

There are several hypotheses as to why SARS-CoV-2 seems to produce hematological problems, but further investigation is needed to establish the exact molecular mechanism. However, various mechanisms have been proposed in Figure 2 [35]. Firstly, the interaction of SARS-CoV-2 and the ACE2 receptor might result in hematological disorders. A recent analysis of bioinformatics data indicates that the ACE2 receptor mediates the SARS-CoV-2 cell entrance mechanism, and SARS-CoV-2 penetrates the cells by using the S protein to recognize and bind to ACE2. This leads to changes in the S1 domain and exposure of cleavage sites for proteases like TMPRSS2 or furin, which cleave the S protein between S1 and S2, allowing the virus to merge with the cell membrane and enter the cell. After accumulating inside the cell, the virus undergoes a similar replication cycle to other RNA viruses while its expression is not limited to the alveolar cells of the lungs but also extends to a variety of various tissues (including intestinal smooth muscle, heart, liver, kidneys, and the endothelium of blood vessels) [31] and lets the virus hematogenously spread throughout the body via the circulatory system to directly infect blood vessels causing complications of blood including thromboembolic events and coagulation disorders [35,36]. Due to a vicious cycle, M1 macrophages create chemical mediators that decrease the density of enzyme ACE2 receptors in blood vessels due to viral endocytosis, and levels of angiotensin II rise, causing vascular, proinflammatory, and profibrotic consequences [37]. Additionally, this mechanism serves as a positive feedback loop to increase ACE2 production. Repetitive stimulation of these cycles enhances the spread of an infection and consequently raises levels of angiotensin II and can also take part in the pathophysiology of COVID-19 by causing heightened inflammation, vasospasm, and fibrosis [36,38,39]. A prothrombotic phenotype is generated due to the enhanced activation of inflammatory cytokines IL-1 and IL-6 by activated M1 phenotype macrophages (Interferon-7) and excessive Ang II activity leading to increased permeability, endothelial cells activation, and co-expression of adhesion molecules [40,41]. In addition, this is observed via an increase in the synthesis of various chemicals (such as tissue factor, plasminogen activator inhibitor factors I, and von Willebrand factor (VWF)) that take part in hemostasis, which causes hemostatic alteration that leads to endothelial inflammation, both prothrombotic and pre-adhesive [33,42]. Since SARS-CoV-2 infects endothelial cells directly, this indicates persistent inflammation in blood vessels throughout the body that ultimately leads to endotheliitis with symptoms including blood clots, brain fog, shortness of breath, and fatigue.

Autopsy studies have also been conducted to further comprehend the hematological system involvement in COVID-19; however, in an autopsy study of 26 deceased COVID-19 patients by Lax and colleagues, blood clots in the tiny arteries caused a disorder called thrombotic microangiopathy, which was found in 11 of the 26 patients, leading to damage to vital organs like the kidneys and lungs. The study also found that many patients exhibited hematological problems such as anemia and leukocytosis, as well as high levels of D-dimer, a protein fragment that indicates the existence of blood clots [43]. Another study also investigated autopsy specimens from 12 COVID-19 patients. The investigation revealed that all 12 patients had multiple organs affected, including the heart, lungs, and kidney, by microvascular thrombosis or blood clot development in small blood vessels. Hematological problems such as thrombocytopenia and lymphopenia were also common among the patients, and the study showed that many of them had increased levels of D-dimer as well [26].

All these findings and hypotheses may have clear implications for transmission prevention measures, especially for hospitalized COVID-19 patients. There are currently few studies on how hematological disorders affect transmission precautions for COVID-19 patients who are hospitalized. However, it has been observed in a study that patients with hematological complications might have an increased chance of developing COVID-19 because of their increased exposure to healthcare professionals during their prolonged hospitalizations. Data from 193 COVID-19 patients hospitalized in Italy revealed that those with hematological diseases had to stay in the hospital longer and were more likely to acquire healthcare-associated infections than those without hematological disorders. According to the study’s authors, individuals with hematological illnesses may require additional transmission precautions like isolation and personal protective equipment (PPE) for healthcare staff [44]. Current recommendations state that a minimum of two negative RT-PCR tests on respiratory specimens for SARS-CoV-2 taken 24 h apart should be used at the moment of discharge of hospitalized COVID-19 cases with hematological disorders [45].

## 5. COVID-19 and Pre-Existing Hematological Complications

In general, patients with COVID-19 who also suffer from co-morbidities have a lower chance of a successful recovery. This could potentially impact the management of patients with pre-existing hematological disorders. Cancer patients have an increased vulnerability to developing infections, although it is still unknown whether hematological malignancies overall are more susceptible to contracting SARS-CoV-2 than the general population. The prevalence of blood disease symptoms was particularly high in cancer patients and in COVID-19 patients in general. For example, in one study, 53 hospitalized patients with COVID-19 had active hematologic malignancies, including lymphoma, leukemia, or multiple myeloma, and researchers found that 34 (64%) out of 53 patients required intense care, and the rate of mortality was 25%. The study also found that the mortality rate was higher in the infected population with lymphoma in contrast to leukemia or multiple myeloma in infected cases [24]. A retrospective study with 1392 patients with cancer and COVID-19 has also focused on a comparison of inpatients with cancer and outpatients with cancer history. The study observed that 18.3% of the patients had leukopenia and 19.5% had thrombocytopenia. The study also found that COVID-19 cases with hematological malignancies had an increased risk of leukopenia and thrombocytopenia compared to tumor cancer patients while also observing that the mortality rate is higher in thrombocytopenia patients as compared to patients without these blood abnormalities [46].

### 5.1. Coagulation Manifestations: Disseminated Intravascular Coagulation

There is growing evidence that COVID-19 infection causes hemostatic manifestation. Venous thromboembolism (VTE) is more common in critically ill individuals due to immobility, systemic inflammation from critical illness (like severe acute pancreatitis), desiccation, endothelial stasis, and dysfunction [47]. Some patient-associated risk factors for VTE development are metabolic syndrome, e.g., diabetes mellitus, arterial hypertension, obesity, coronary heart disease, history of VTE inherited thrombophilia, and peripheral artery disease [48,49]. It is known that infections in critically sick patients can cause disseminated intravascular coagulation (DIC), a condition where the blood clots abnormally throughout the body and leads to damage endothelium, activation of intravascular coagulation, activation of neutrophils, and life-threatening complications [50].

**Figure 3:**
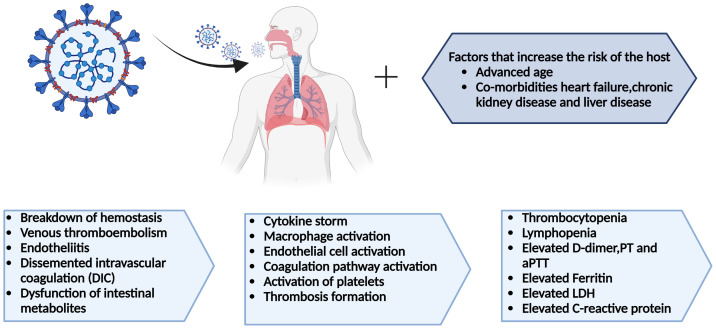
COVID-19-associated hematologic manifestations like changes in blood cell counts (lymphopenia, neutrophilia, thrombocytopenia), coagulation disorders (DIC, increased risk of VTE), and abnormal levels of D-dimer, CRP, LDH, and clotting factors. These issues show the complex connection between the virus, immune response, and blood clotting.

Patients with DIC have a condition called sepsis-induced coagulopathy (SIC), which occurs earlier in the patient and is less severe. These changes occur sequentially, and if the cause is left untreated, SIC becomes DIC [51]. In 2009, the International Society on Thrombosis and Hemostasis (ISTH) published a scoring system showing that coagulopathy caused by COVID-19 meets the criteria for SIC or DIC [52]. Moreover, there is also an agreement that coagulopathy in COVID-19-related DIC resembles thrombotic microangiopathy, which complement activation damages endothelial cells, and that COVID-19-associated DIC is thought to be caused by a virus that causes inflammation, which causes bleeding all over the body [53,54]. Figure 3 outlines the COVID-19-associated hematologic manifestations.

### 5.2. Hemostasis Disorders: Thromboprophylaxis and Thrombosis

Hemostatic disorders associated with COVID-19 infection are increasingly being identified. An increasingly clear clinical picture of COVID-19 infection shows an increased chance of venous thromboembolism (VTE), especially in severe illness with arterial thrombotic, deep venous thrombosis (DVT) instances as mini stroke [55,56,57]. The exact thrombotic issues had to be recorded with either a contrast-enhanced computed tomography (CT) scan or a Doppler ultrasound, and scans were requested upon the treating physician’s decision. Cui and colleagues from China analyzed 81 COVID-19 patients, and 25% were found to have VTE, while thromboprophylaxis details were not given [58]. Among 388 Italian patients in a retrospective cohort analysis, 61 (16%) were brought to the ICU, 327 (82%) patients were treated in normal medical wards, and 78% were given thromboprophylaxis. The overall incidence of thromboembolic instances occurred in 21 percent of patients; only eight infected cases received a diagnosis of overt DIC, but seven did not survive. D-dimer levels increased rapidly in the non-survivors, which is consistent with recent studies [59]. Recently, a Dutch analysis of 184 critical ICU cases with COVID-19 infection receiving thromboprophylaxis showed at least 31% of thromboembolic events. The predominant thrombotic consequence was pulmonary embolism (81%). Independent predictors of thrombotic events were prothrombin times less than 3 s and aPTT less than 5 s (hazard ratio (HR) 4.1, 95% CI 1.9–9.1) [56]. Another study found that out of 75 patients hospitalized in the ICU infected with COVID-19, 46.6% experienced thromboembolic events [60]. Similarly, postoperative thromboprophylaxis for this group of patients has very sparse indications. The initial COVID-19 flow in New York City had 11,249 patients, and 1.7% of them developed venous or arterial thromboembolism (VTE or thromboembolism) [61]. As shown in this trial, preventive anticoagulation with enoxaparin or rivaroxaban reduced coagulation complications by 46% [61]. On the other hand, patients with COVID-19 pneumonitis may bleed and clot simultaneously when treated with anti-coagulants, making this condition pathophysiologically distinct [62]. Major bleeding occurred in 1.73% of patients within 90 days of discharge, while only 13.2% of the population received thromboprophylaxis [61]. Wichmann and coworkers found that deep venous thrombosis (DVT) was present in 58% of autopsied cases involving 12 patients. Despite the presence of pulmonary embolism (PE) in 4 out of 12 patients, microthrombi were frequently observed in the small lung arteries [63]. Diffuse microvascular thrombi have been found in a variety of organs in autopsy studies performed in China [64]. Prominent PE was detected in 4 of 21 COVID-19 patients in another autopsy group, and alveolar capillary micro-thrombosis was detected in 5 of 11 patients (45%). Thrombotic microangiopathy of the glomerular capillaries was present in three of them [65]. COVID-19 has also been associated with abnormal blood clotting tendencies. Cases of pulmonary embolism (PE) demonstrated a more rapid increase in thrombin generation, as seen in the CAT (calibrated automated thrombogram) assays conducted upon hospital admission [66].

### 5.3. Thrombocytopenia

In moderate COVID-19 infection, Prothrombin Time (PT) and activated Partial Thromboplastin Time (aPTT) may be ordinary or mildly prolonged, but they will be significantly prolonged in severe illness [67]. Platelet counts range from normal to mildly elevated in mild disease and markedly low in severe disease [68]. D-dimer and fibrinogen increases, on the other hand, are associated with the severity of the illness [28]. Some of these findings are prognostically important. Despite this, most data come from looking back at previous cohorts or conducting subgroup analyses. D-dimer 0.5 μg/mL was more common in very ill patients and affected 46.4% (260) of 560 patients among 1099 Chinese patients with COVID-19. Thrombocytopenia (platelet count < 150 × 10^9^/L) was observed in 315/869 (36.2%) patients, and individuals with severe infection showed a remarkable decrease [69]. Other descriptive studies from China with 99 hospitalized patients showed that 36%, about one-third of the infected cases, had a high level of D-dimer, and 12% of the patients had acquired thrombocytopenia [18]. Thrombocyte levels often decrease in COVID-19 patients admitted to the ICU. Thrombocytopenia has been considerably lower in infected cases of serious infection, according to a meta-analysis of 1779 cases infected with COVID-19 from nine separate trials (mean difference in thrombocytes: 31 × 10^9^/L; 95% CI = 35–29 × 10^9^/L). More than five times the probability of critical SARS-CoV-2 infection has a correlation with having a low platelet count (OR = 5.1; 95% CI = 1.8–14.6). The etiology of thrombocytopenia is complex, but potential contributing factors include endothelial cell injury from ventilatory dysfunction and stimulation of platelets, abnormal platelet dispersion of megakaryocytes in the bronchial blood vessels, and affected myelotoxicity from thrombocytopenia and SARS-CoV-2 infection [11]. Another retrospective study including 41 patients confirmed that those requiring hospitalization in the ICU had increased PT and D-dimer levels than those who did not. Only 5% of the patients had severe thrombocytopenia (<100 × 10^9^/L) [17]. In a retrospective study, Cui et al. analyzed that D-dimer threshold levels predict VTE with a susceptibility of 85.0%, specificity of 88.5%, and an NPV (negative predictive value) of 94.7% among 81 patients [58]. Similarly, a retrospective analysis of 1449 COVID-19-infected cases in China found that those who ultimately passed away had higher mean and median values for D-dimer, PT, and aPTT. Increased mortality was associated with rising levels of D-dimer, fibrinogen, and platelet counts [70]. A case series study of 30 diagnosed COVID-19 cases showed that patients whose platelet counts peaked had worse clinical outcomes [71]. Furthermore, the peak platelet-to-lymphocyte ratio (PLR) value during treatment also played a significant role in determining how long a patient stayed in the hospital. It was suggested that increases in platelet count and long stays in the hospital have been linked to the cytokine storm.

### 5.4. Lymphopenia

SARS-CoV-2 illness is linked with decreased CD4+, CD8+ T-cell, NK-cell, and B-cell lymphocyte counts in critically ill patients and is thought to occur through a separate mechanism [72]. It is known that SARS-CoV-2 enters host cells by attaching its spike protein to the ACE2 receptor, thereby enabling the virus to invade the cell and replicate, causing SARS-CoV-2 infection. Lymphocytes also express these receptors on their surface. Therefore, viruses may trigger lysis by binding directly to these cells [35]. Multiple inflammatory cytokines are produced and released in response to infection. Lymphocyte shrinkage and apoptosis caused by this powerful cytokine activity can reduce lymphocyte regeneration [35,36]. Moreover, CD4+ T cells also have an important function as immunological modulators, including the suppression of inflammation [73]. Therefore, lymphopenia may play a role in the chain reaction that leads to excessive inflammation. Disease progression is influenced by natural killer cells and reduced CTLs (cytotoxic T lymphocytes) because they are essential for resisting viral infection [74]. Lymphopenia has been observed in a considerable quantity of COVID-19 patients, particularly T cells in critically ill populations, but is generally reversible even after recovery from COVID-19 and is thought to be a risk for increased mortality, seriousness, and worse prognosis [75]. Lymphopenia also affects CTLs and NK and B cells and has been linked to the disease severity [76]. 

Transient lymphopenia has frequently been studied in various viral flu infections, including RSV (respiratory syncytial virus), H3N2v virus, and SARS [77]. However, the number and duration of lymphocytes in COVID-19-infected patients appear to be more severe and selective for T-cell lineage, and they are also found to be prolonged compared to other viral respiratory illnesses [78]. Lymphopenia was observed in 70.4% (19/27) of the most severely ill patients at a hospital in Wuhan during the early stages of the COVID-19 outbreak, with T cells declining more than B lymphocytes. Surprisingly, when symptomatic disease was treated, CD4+ and CD8+ T cells were considerably enhanced, whereas B lymphocytes and NK cells were not. This suggests a possible correlation between clinical and immunological recovery, which can be assessed by an increasing peripheral CD4 and CD8 count [79]. In another study, the CD4/CD8 ratio remained within the normal range, but CD8+ T cells improved more after treatment [80]. According to a study of the hematologic features of infected cases hospitalized at China’s Wuhan Union Hospital, patients with critical illness had a low count of lymphocytes and a higher NLR (neutrophil-to-lymphocyte ratio) than those with severe or moderate illness. The average number of lymphocytes in this study was reduced in patients with severe disease (0.54 × 109/L) compared to those with intermediate disease (1.2 × 109/L). In addition, higher NLR has also been linked to the intense inflammatory response observed in sepsis and subsequent lymphocyte loss due to SARS-CoV-2 [21]. In another study conducted at five hospitals in Wuhan, China, patients who died of COVID-19 had lower median lymphocyte counts than survivors [70]. In Wuhan, other studies have found that people who did not survive COVID-19 had longer severe lymphopenia compared to those who survived, and the rate of lymphocyte decline remained until deceased. Surviving infected cases had the lowest lymphocyte counts on day 7 but then saw an improvement during their hospitalization. Patients with persistently low lymphocyte counts were found to have a higher risk of mortality [30]. As a result, monitoring lymphocyte counts can be useful in assessing disease severity and predicting consequences in COVID-19-infected cases. Tan et al. developed a model to classify disease severity and predict prognosis based on the lymphocyte percentage (LYM%) in COVID-19 patients. The model suggests that patients with an LYM% less than 20% in the second week after symptom onset have a favorable outcome and make a full recovery rapidly, while those with an LYM% greater than 20% are classified as severe cases. After the third week of experiencing symptoms, infected cases with an LYM% less than 20% are likely to recover, those with an LYM between 5% and 20% are susceptible to decompensation, and those with an LYM% less than 5% are considered to be in critical condition, necessitating intensive care. Certainly, COVID-19-infected individuals often exhibit lymphopenia, which is considered a crucial indicator for prognosis and the severity of the illness.

The impact of COVID-19-induced lymphopenia on the immune system has been studied further. Zheng et al. investigated the immune-mediated features of leukocytes in peripheral blood from 16 COVID-19 patients in China, Kunming, and concluded that COVID-19 impaired the CD4 T cells’ functions and caused excessive activation; consequently, lymphopenia caused by CD8+ T-cell depletion may compromise the immune reaction to SARS-CoV-2 in severely ill patients [81]. Qin et al. conducted a group analysis of 452 individuals with COVID-19 infection in Wuhan and found that their immune responses were dysregulated. Researchers discovered that severe cases had decreased monocyte, eosinophil, and basophil percentages while increasing neutrophil and leukocyte counts. Analysis of lymphocyte subpopulations in 44 infected COVID-19 cases also showed a reduction in B, T, and NK cells, with a marked difference between severe and non-serious cases. In severe instances, SARS-CoV-2 was found to reduce T-cell counts to close to 50% of the lower limit of normal (461.6/L compared to 663.8/L; *p* = 0.027), demonstrating that T cells were more affected than other cell types. Subset analysis of T cells revealed that those with COVID-19 had decreased numbers of both helper T (CD3+ and CD4+) cells and suppressor T (CD3+ and CD8+) cells. These results suggested that COVID-19 may cause immune system malfunction by directly harming lymphocytes during the acute infection stage [27]. A high incidence of lymphopenia, ranging from 67–75%, has been consistently reported among COVID-19 patients who have a severe illness; it may correlate with an increased quantity of cytokines such as IL-6, IL-10 or TNF (tumor necrosis factor) [82,83]. These cytokines and TNF may have a direct impact on the T-cell population [84] and might have an indirect impact on other types of cells, including neutrophils and dendritic cells [85,86].

Therefore, the reduction in the number of T lymphocytes, especially in the periphery, is a major hallmark of many people with severe disease, but the mechanisms of lymphopenia in COVID-19 remain incompletely known. Why lymphopenia is more prevalent in T cells, and possibly only CD8+ T cells, is still unknown. Lymphopenia has been shown to increase the activation of T cells and proliferation in animal studies [87]. Future research should emphasize elucidating the role of lymphopenia in COVID-19 patients, as treatments like IL-7 may be useful in reducing T-cell hyperactivation and, potentially, immunopathology.

### 5.5. Neutrophilia

COVID-19-related disruption of immune regulation contributes to various alterations in the immune system, including neutrophils. Additionally, neutrophilia might be related to an overlying bacterial infection, which is more common in severely ill patients [71]. Certainly, COVID-19 can cause hyperinflammatory responses and overproduction of cytokines, which activate and attract immune cells like macrophages, monocytes, and neutrophil infiltration into lung tissue. These cells can worsen tissue damage and increase the severity of the illness [73]. A study examined the immunological responses of 13 COVID-19 patients and observed that the illness has elevated cytokines proportion with IL-6, IL-10, and TNF-alpha, along with enhanced monocytes and neutrophil counts. As a result, the hyperactive reaction and subsequent cytokine storm in COVID-19 may contribute to the excessive recruitment and neutrophils stimulation and monocytes, which can lead to lung damage and other problems like MIS (multisystem inflammatory syndrome) [88]. Another study examined cytokine levels and COVID-19 severity. Patients with serious COVID-19 illness had high IL-6, IL-8, and TNF-α levels, along with higher neutrophil and monocyte counts. The study reveals that the cytokine storm, neutrophil, and monocyte activation may contribute to COVID-19 progression and severity [89]. Autopsy of lung tissue samples from Chinese COVID-19 patients examined neutrophil infiltration of lung capillaries and fibrin deposition in inflamed capillaries generate acute inflammation causes widespread alveolar destruction and ARDS and suggest that neutrophil infiltration may contribute to the acute injury of lungs in COVID-19 severe cases [90]. Another Italian study observed extensive alveolar damage, hyaline membrane development, and microthrombi in the pulmonary vasculature in COVID-19 lung tissue samples, suggesting severe endothelial injury [91]. Neutrophils have been identified as an indicator of respiratory symptom severity and poor outcomes in COVID-19. Neutrophil extracellular traps (NETs) are the main mechanism by which neutrophils induce inflammation in various organs and play a crucial role in damaged organs [92].

NET is a network of chromatin and antimicrobial proteins in the extracellular space, such as cathelicidin, calprotectin, myeloperoxidase (MPO), neutrophil elastase (NE), and others. The composition of NETs can vary and is influenced by the stimulus that initiates their release from neutrophils to trap and kill pathogens. NETs are antimicrobial, but they also cause a lot of damage to the host’s tissue and make inflammation worse in many acute and chronic illnesses, including lung diseases [93]. Neutrophil elastase activation also participates in the excessive formation of NETs in the inflammation cascade, which can lead to microthrombosis, tissue damage, and organ failure involving the lungs, heart, and kidney, and contributes to a diversity of infections pathogenesis, including SARS-CoV-2. It mainly occurs due to dysregulated signaling during severe immune response in which NETs activate macrophages to generate proinflammatory cytokines IL-1β and TNF-α, and this further promotes NET formation. Moreover, IL-1β generates IL-6, which sticks to soluble IL-6 receptors released by neutrophils and causes signaling associated with a proinflammatory state [94]. Individuals with severe COVID-19 had raised levels of NETs in their blood, thrombi, and lungs. This suggests that neutrophils and NETs might be essential in the pathophysiology of COVID-19, and their association has worse outcomes in various inflammatory and infectious illnesses [95]. Neutrophilia infiltration activation has been linked with COVID-19 disease development and a higher risk of emerging ARDS and mortality. Several studies have established that critical COVID-19 patients’ blood and tissues have elevated levels of neutrophils compared to those who have mild illness. The NLR (neutrophil-to-lymphocyte ratio) is a simple marker of systematic inflammation and has also been described to predict critical illness and mortality in infected people with SARS-CoV-2 [96]. Ding et al. showed a case–control analysis of 72 COVID-19-infected patients to determine whether there was a connection between the length of hospital stay and changes in hematological blood parameters. Of the 72 patients, a total of 39 (54.2%) developed lymphopenia and 20 (27.2%) developed leukopenia, while 15 (20.8%) were classified as severe instances and 57 (70.2%) were classified as mild cases. Lymphocyte counts often decreased in severe patients, while leukocyte and neutrophil counts and the neutrophil-lymphocyte ratio (NLR) markedly increased in mild-infected patients. Platelet counts in non-severe patients were found to rise gradually throughout the course of the study. They also found that NLR was positively correlated with hospital stay duration beginning on day five, suggesting that NLR was involved in predicting the prognosis for COVID-19 patients [97]. Recent research has suggested that NLR can be utilized to indicate the severity of SARS-CoV-2 illness in its early stages. The prognosis for illness progression was best predicted by NLR, followed by patient age, in a study observed in Beijing, China. Infected people with NLR 3.13 and age ≥ 50 years had a 50% chance of developing critical illness, while individuals with NLR 3.13 and age < 50 years had a low incidence of 9.1% [98]. A similar finding from a retrospective cohort study conducted at Wuhan University indicated that COVID-19-hospitalized patients with a greater NLR had a significantly increased risk of mortality from any cause [99]. A review of 38 studies was conducted to assess the significance of the NLR in expecting severity of the infection and mortality in COVID-19 infected cases. The meta-analysis revealed that elevated NLR values on admission were related to increased chances of disease severity and fatality. This suggests that NLR is a useful biomarker for predicting the diagnosis of COVID-19 patients. Also, the difference in NLR values was greater between surviving and non-surviving patients compared with critically ill and non-critically ill patients. Patients with high NLR levels on admission had approximately double the risk of death compared with patients with normal NLR levels. Age, sex, diabetes mellitus, arterial hypertension, and cardiovascular diseases did not influence these relationships [100]. Certainly, the measurement of NLR from routine serum testing is a simple and effective way for clinicians to recognize COVID-19-infected populations that have a higher chance of developing serious sickness and death, which may help in prioritizing treatment, allocating medical sources and classifying patients into higher levels of attention [99].

## 6. Biomarkers

**Figure 4:**
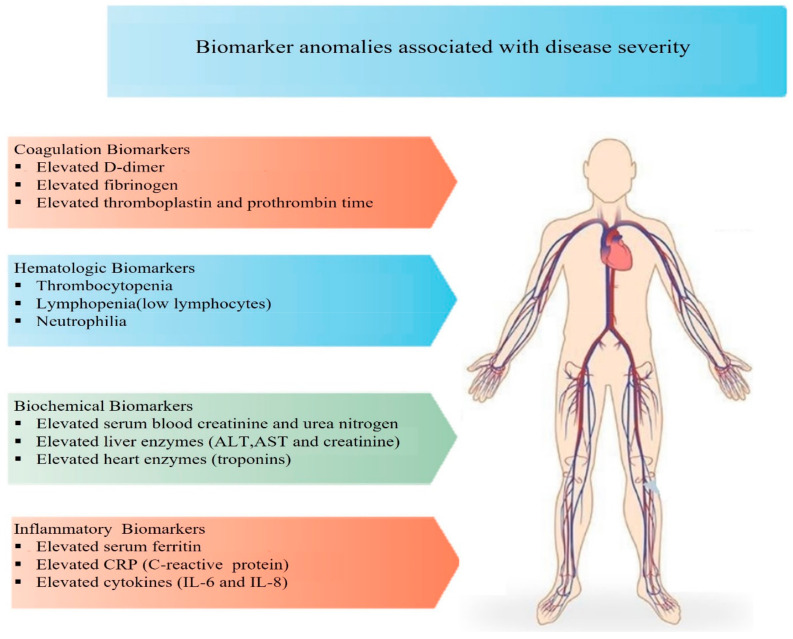
In severe COVID-19 cases, biomarker anomalies play a critical role in assessing disease severity and prognosis. These biomarkers include elevated levels of inflammatory markers, hematological biomarkers, and altered coagulation parameters, as well as other biomarkers indicating tissue damage and organ dysfunction.

SARS-CoV-2 may involve a wide range of COVID-19 severity, from asymptomatic to life-threatening infections, and its epidemic has directed an urgent need to identify serum biomarkers that can indicate illness severity. During the incubation period, leukocytes and lymphocytes remain unaffected, but in the second phase of the disease, the virus can cause viremia, leading to pneumonia and potentially causing respiratory failure [17]. In severe cases of COVID-19, as described earlier, lymphocyte counts are reduced while levels of inflammatory markers such as CRP (C-reactive protein), ferritin, and ESR (Erythrocyte Sedimentation Rate) are increased. High levels of ALT/AST, D-dimer, and LDH (lactate dehydrogenase) can also be detected in the blood [101], as shown in Figure 4.

CRP, a biomarker produced by the liver and induced by IL-6, is commonly elevated in severe cases of COVID-19, making it a sensitive marker of inflammation and tissue damage. Several studies suggest that serum CRP levels serve as a dependable marker of both the presence and intensity of the SARS-CoV-2 sickness. Several studies have examined that serum CRP levels are a dependable marker of the existence of SARS-CoV-2 disease severity [102]. In Wuhan, China, a survey discovered that there was a rise in the advancement of the disease among patients with CRP levels exceeding 41.8 μg/mL [25]. In an alternative study, CRP intensity and pulmonary abnormalities observed via CT scan were studied, and the results showed that in the initial phase of COVID-19, elevated CRP levels correlate with both the size of the lung lesion and the extent of disease severity [103]. A different investigation assessed the relationship between CRP levels and CT intensity scores, which assign a numerical value based on visual examination of many lung lobes. The study revealed a direct association between CRP levels and CT outcomes. The authors suggest that CRP may be a more reliable predictor than CT scans in earlier stages of infection, when CT outcomes may not express substantial differences [104].

LDH (lactate dehydrogenase) is a main enzyme found in almost every tissue in the body, including the blood cells, heart, lungs, liver, muscles, and kidneys, and plays a crucial role in cellular metabolism to convert pyruvate to lactate and vice versa [101]. LDH is known as a biomarker because when its level becomes elevated in the blood, it signals the occurrence of tissue injury or infection. When tissues or cells become damaged, they express LDH, leading to higher LDH levels in the serum. Therefore, an increase in LDH is often observed in various diseases such as liver disease, heart disease, cancer, and infections like COVID-19, and it is proposed that in COVID-19 individuals, elevated LDH levels are considered to be an indication of severity and worse prognosis [105]. As mentioned earlier, elevated LDH levels are often associated with the intensity of SARS-CoV-2 sickness. These levels are supposed to be connected to tissue damage and inflammation, with elevated levels of LDH being associated with this condition [101]. A report evaluated a series of cases and established that high LDH levels upon admission, advanced age, CRP, and lymphopenia were linked to the requirement for an intensive care unit (ICU) [105]. Martinez and colleagues compared the levels of certain compounds in severe COVID-19 individuals and those with ARDS (infected group) to non-ARDS (moderate group) pneumonia. The results pointed out that infected COVID-19 individuals had an elevated range of CRP, LDH, and ferritin, which exhibited a marked increase in critical cases than in mild to moderate ones. Furthermore, severe cases showed increased systemic inflammation, as indicated by elevated leukocyte, LDH, ferritin, IL-6, and TNF-α levels a week after admission. The study implies that elevated levels of inflammatory markers and a decrease in certain protective factors may contribute to an elevated rate of fatality in COVID-19-hospitalized cases. The best markers for predicting disease severity were found to be TT, ferritin, and LDH, whereas D-dimer MMP-9 significances have notably elevated in critically ill patients during hospitalization [106]. Although further research is needed, LDH has been related to an unfavorable outcome in COVID-19. D-dimer is a protein fragment that is generated when blood clots are detected in the body, and this protein is called fibrin. As a result of its lysis, D-dimer is released into the blood, signifying the initiation of fibrinolysis and coagulation [101]. D-dimer is an additional significant biomarker often used to examine blood clotting complications and as a potential prognostic factor and diagnostic tool for COVID-19 infection severity. A preliminary study that examined coagulation indicators in patients with COVID-19-associated pneumonia found that those who died from COVID-19 had considerably increased levels of D-dimer upon hospital entry, and these levels remained elevated later in the disease in all fatal cases [107]. Kaftan et al. conducted predictive levels of biomarkers in individuals with COVID-19. In addition, D-dimer is a fibrin degradation test used for diagnosing thrombotic diseases. Previous studies have shown that higher levels of D-dimer are associated with severe community-acquired pneumonia and chronic obstructive pulmonary disease. In COVID-19 cases, D-dimer levels increased by 1 μg/mL are a risk factor for mortality, and levels greater than 2.0 μg/mL upon hospitalization are linked with disease severity and mortality. D-dimer might be a useful indicator for managing COVID-19 patients [108]. Even after adjusting for age or sex, an increased level of D-dimer remained an important factor regardless of the presence of underlying diseases.

Ferritin not only exhibits the function of storing iron but it is also recognized as an indicator or biomarker of an acute phase reactant that responds to inflammatory stimuli, as its level can increase in response to cytokines like IL-6 that are produced during infection or an inflammatory response. The H subunit of ferritin, which is produced in response to inflammatory stimuli and may operate as an immunological regulator with both inflammatory-promoting and immunoregulatory properties, is one of two subunits that make up ferritin [109]. Elevated ferritin levels in COVID-19 cases are supposed to be linked to cytokine storms, which are an increased immune response that can occur in serious COVID-19 patients [110]. The cytokine storm is considered to be the high amount of cytokines associated with inflammation released into the body, which can cause tissue or organ damage and can lead to the development of serious complications such as ARDS, so ferritin levels are high in response to infection or inflammation. Studies have shown that ferritin also serves as a useful biomarker, and its elevated level is linked with disease intensity and poor consequences in individuals with COVID-19 sickness [111]. A report in Wuhan, China, retrospectively evaluated a cohort of infected cases and initiated that high ferritin levels, along with several further biomarkers such as CRP, neutrophilia, lymphocytopenia, and D-dimer, LDH, were markedly linked with a greater risk of developing ARDS. However, the study did not find an association between ferritin and illness mortality [19]. Another study showed that highly elevated levels of serum ferritin (>3000 ng/mL) in 8% of our population upon admission led to 13% being shifted to ICU, and 12% of patients had expired along with high levels of CRP, D-dimers, and low lymphocyte counts, which were determined an autonomous serious chance for disease acuteness in COVID-19 cases. Additionally, highly elevated ferritin showed an independent correlation with hemoglobin counts with a poor outcome [111]. Besides ferritin and IL-6, other commonly used inflammatory biomarkers are important in monitoring COVID-19 patients. Troponin is a protein present in heart muscle cells, and its elevation in the serum shows heart muscle damage. Studies showed that elevated troponin levels in COVID-19 patients are associated with disease severity and increased mortality [112].

Extra-thyroidal procalcitonin levels increase during severe bacterial infections due to endotoxins and cytokines, while viral infections lead to the downregulation of procalcitonin. In uncomplicated COVID-19 cases, procalcitonin levels are usually within the reference range. However, elevated levels may indicate both a bacterial infection and the progression of serious illness [29]. Procalcitonin levels slightly higher in the serum may help to distinguish between COVID-19-infected and COVID-19-non-infected individuals, and the increase in procalcitonin is more significant in critically ill patients [113]. Xu et al. showed that elevated levels of procalcitonin are closely associated with CRP and NLR among COVID-19 individuals admitted to the hospital with high mortality [114]. Kidney and liver function markers have been found to be important indicators of serious illness and fatality in COVID-19 cases. Studies have shown that elevated levels of enzymes like ALT, AST, and creatinine, or the progression of acute renal infection, are significantly connected with a greater risk of death in COVID-19-infected cases. In addition, a systematic review has demonstrated that acute renal infection is an indicator of poor clinical outcomes in COVID-19 cases [115].

## 7. Prevention and Management

The management of hematological complications in patients with COVID-19 is essential for enhancing outcomes and, ideally, mitigating the occurrence of serious complications. The COVID-19 virus has been linked to an increased susceptibility to clotting disorders such as DIC (disseminated intravascular coagulation), DVT (deep vein thrombosis), and PE (pulmonary embolism) [116].

COVID-19 pathogenesis is linked to both the infiltration of lung epithelial cells by SARS-CoV-2 and the immune response of the host against the virus [117]. An uncontrolled systemic inflammatory reaction triggered by the release of excessive proinflammatory cytokines stands out as a prominent feature of severe acute respiratory distress syndrome (ARDS) and multiple organ failure—the primary causes of death in COVID-19 cases [117]. In addition to inflammation, patients with COVID-19 might exhibit indications of hypercoagulability, characterized by notable increases in fibrinogen levels and D-dimers, and at later stages of the illness, it might undergo disseminated intravascular coagulation (DIC) [118,119].

Elevated D-dimer levels and the DIC diagnosis are linked with worse prognosis and death [119]. Furthermore, accumulating evidence confirms that there is an increased frequency of arterial and venous thrombosis in COVID-19 cases, and these clotting events are tied to elevated rates of mortality [118]. Consequently, the prevention of thrombosis plays a critical role in the comprehensive clinical management of hematological complications of COVID-19 cases patients. In brief, the proper management of COVID-19 patients with hematological complications involves four pivotal factors, each bearing paramount significance:

(I) Early Diagnosis and Follow-up of Disseminated Intravascular Coagulation (DIC): A crucial step involves prompt early diagnosis and subsequent monitoring of DIC, a complex hemostatic disorder often witnessed in COVID-19 patients. Using the scoring system from the International Society on Thrombosis and Hemostasis (ISTH), which considers factors including platelet count, prothrombin time (PT), fibrinogen levels, D-dimer concentrations, as well as antithrombin and monitoring of protein C activities, not only provides thorough evaluation but also insight into the prognosis. This smart method helps direct personalized critical care actions, ultimately creating a more precise and powerful supportive plan [120].
(II) Identification of High-Risk Patients, Irrespective of Clinical Setting: It is important to identify individuals at elevated risk for thrombotic events, whether they are hospitalized or ambulatory. This keen awareness is important for addressing potential complications early and tailoring treatment to the infected person’s needs. By recognizing those vulnerable to thrombotic complications, healthcare providers can proactively navigate the clinical management of COVID-19 patients with hematological complications in advance, thereby potentially minimizing adverse outcomes [121].
(III) Optimization of Thromboprophylaxis Regimen and the Role of Low Molecular Weight Heparin (LMWH): An important part of taking care of patients is making sure we obtain the right balance in how we develop thromboprophylactic strategies. In this regard, low molecular weight heparin (LMWH) comes in as a top-notch treatment. Not only is LMWH good at reducing thrombotic risks, but it also has some anti-inflammatory qualities that could be extra helpful for COVID-19 patients with hematological abnormalities. To make the most of LMWH, it is crucial to pay close attention to how much is given and when—that is how we can make sure it works its best and helps manage this complicated clotting situation in the body [122].
(IV) Integration of Antithrombotic Treatments in the “Immunothrombosis” Paradigm: Figuring out the complicated interaction between immunological and thrombotic pathways, as observed in COVID-19, requires a creative strategy. Bringing an extra tool such as antithrombin and recombinant thrombomodulin assumes a key role in modulating the interaction between “immune response and thrombosis” (Immunothrombosis process. These tools synergistically enhance the anti-inflammatory and anti-coagulant aspects of treatment, showing how treatment plans are becoming better at matching the changing ways the disease affects the body [123].

Managing COVID-19 patients with hematological complications requires a carefully planned combination of actions. It is crucial to quickly diagnose the infection and conduct blood tests like peripheral blood smear, platelet count, PT, aPTT, fibrinogen, and D-dimer levels upon admission, evaluating the risks, using smart ways to prevent thromboprophylaxis (blood clots), and using treatments that specifically fight clotting with regular monitoring for critically ill patients [116]. Venous compression Duplex scans are suggested for ICU patients upon admission and periodically thereafter to detect and prevent deep vein thrombosis (DVT), alongside vigilance for signs of pulmonary embolism (PE) [116,124]. Diagnosis of disseminated intravascular coagulation (DIC) does not always warrant anticoagulation unless clotting is present. Thromboprophylaxis, using agents like LMWH, is recommended for nearly all hospitalized COVID-19 patients, with dosage adjusted as needed. Prophylactic treatment should persist throughout hospitalization, and continued use after discharge might be considered for high-risk cases. Blood clot diagnoses should rely on imaging tests rather than D-dimer levels alone. LMWH is preferred for acute blood clot treatment, and switching medications to LMWH is suggested in cases of drug interactions or ICU admission [125,126]. All these actions come together to create a well-balanced plan that is tailored to deal with the complicated and ever-changing way that COVID-19 affects the body’s blood clotting process. As knowledge evolves, keeping up to date with research and guidelines is vital for medical professionals.

## 8. Conclusions

In conclusion, COVID-19 affects multiple systems in the body and has significant hematologic manifestations. It leads to a state of hypercoagulability, possibly due to immune-mediated factors, and can cause substantial harm. Various laboratory parameters, including D-dimer and LDH, may carry prospective values, although close monitoring and interpreting of these biomarkers can aid in predicting patient outcomes and making informed clinical decisions in developing personalized treatment therapy for serious patients. Thromboprophylaxis is essential to prevent VTE, and early identification of potentially fatal problems such as DIC, PE, and stroke is critical for improving the patient’s health. Diagnostic testing for these abnormalities can help identify high-risk patients and provide timely interventions to improve their clinical prognosis. Overall, a heightened approach is necessary to manage risky and severely ill individuals with COVID-19. Vigilant monitoring of coagulation abnormalities is essential, and proactive measures should be implemented to prevent their occurrence or reduce their adverse impact.

## Figures and Tables

**Table 1:** COVID-19 and the hematological abnormalities.

Reference	Sample Size	Platelets Findings	Coagulation Factors	WBCs
Chen et al. [18]	99	Thrombocytopenia 12%	Decrease in prothrombin time 30% Increase in D-dimer 36% Increase in LDH 76% Increase in CRP 86%	Leucopenia 9% Neutrophilia 38% Lymphopenia 35%
Fan et al. [20]	67 58 non-ICU patients 9 ICU patients	Mild thrombocytopenia 20% ICU patients tend to develop neutrophilia during hospitalization	Raised LDH in ICU patients	Leukopenia 29.2% Lymphopenia 36.9% Both were more prominent in ICU group
Huang et al. [17]	41	NT (Not tested)	Prothrombin time and D-dimer levels are higher in ICU patients	Leucopenia 25% Lymphopenia 63%
Liu et al. [25]	78 11 with disease progression 67 with disease improvement/stabilized	NT	CRP significantly elevated in disease progression; albumin significantly decreased in disease progression group; *D*-dimer was slightly higher in disease progression	No significant difference in WBC in disease progression and stabilization groups. Lymphocytes are slightly lower in disease progression group
Qian et al. [26]	91 9 severe 82 non-severe	Thrombocytopenia 10.9%	Elevated *D*-dimer 24.2% High CRP 53.8% High fibrinogen 24.2% Low albumin 47.3% Higher CRP and *D*-dimer in severe patients than in non-severe patients	Lymphopenia 30.7% Lower WBC count 15.4% Low neutrophil 11% Increased neutrophil 3.3% Higher neutrophils and lower lymphocytes seen in severe patients than in non-severe patients
Qin et al. [27]	452	NT	Higher procalcitonin, CRP, and serum ferritin in severe patients than in non-severe patients	Severe patients had a significantly higher leukocyte, higher neutrophil, higher neutrophil to lymphocyte ratio, lower monocyte, and lower eosinophil than non-severe patients
Wan et al. [28]	135 95 mild 40 severe	Thrombocytopenia 17%	*D*-dimer 0.4 (0.2–0.6) Prothrombin time 10.9 (10.5–11.4) *D*-dimer and LDH were higher in severe patients than in mild patients	Leucopenia 20.7% Lymphopenia 50.4%
Huang et al. [17]	138 36 in ICU 102 non-ICU	Median platelet count was slightly lower in ICU patients than in non-ICU patients	Significantly elevated *D*-dimer, creatine kinase, and lactate dehydrogenase in ICU patients compared with non-ICU patients	Higher WBC and higher neutrophil count in ICU patients than in non-ICU patients Median lymphocyte count was below the normal level
Wu et al. [19]	201	Thrombocytopenia 18.8%	Elevated *D*-dimer 23.3% Significantly elevated for patients with ARDS Prolonged prothrombin time 2.1% Elevated CRP 85.6%	Lymphocytopenia 64% Significantly decreased for patients with ARDS Leukocytosis 23.4% Neutrophilia 34.5% Monocyte elevated 9.1%
Zachariah et al. [29]	50 41 non-severe 9 severe	NT	Procalcitonin levels and CRP were significantly higher in severe patients	Lymphopenia 72% Did not significantly differ between severe and non-severe patients
Zhang et al. [19]	140 82 severe 58 non-severe	NT	Elevated *D*-dimer 43.2% Elevated CRP 91.9% Significantly higher *D*-dimer and CRP in severe patients	Leukocytes decreased by 19.6% Lymphocyte decreased by 75.4% Eosinophil lowered by 52.9% Significantly lower median value for leukocytes and lymphocyte percentage between severe and non-severe patients
Zhou et al. [30]	191 137 survivors 54 non-survivors	Thrombocytopenia 7% More non-survivors showed thrombocytopenia than survivors	Elevated *D*-dimer 32% Elevated in non-survivors compared with survivors throughout the clinical course. High prothrombin time 6%	Lymphocytopenia 40% Leukocytopenia 17% Non-survivors presented with significantly lower leukocytes and lymphocytes than survivors

## Data Availability

Not applicable.
